# T cell receptor β repertoires in patients with COVID-19 reveal disease severity signatures

**DOI:** 10.3389/fimmu.2023.1190844

**Published:** 2023-07-05

**Authors:** Jing Xu, Xiao-xiao Li, Na Yuan, Chao Li, Jin-gang Yang, Li-ming Cheng, Zhong-xin Lu, Hong-yan Hou, Bo Zhang, Hui Hu, Yu Qian, Xin-xuan Liu, Guo-chao Li, Yue-dan Wang, Ming Chu, Chao-ran Dong, Fan Liu, Qing-gang Ge, Yue-jin Yang

**Affiliations:** ^1^ State Key Laboratory of Cardiovascular Diseases, Fuwai Hospital & National Center for Cardiovascular Diseases, Beijing, China; ^2^ Department of Pharmacy and Department of Intensive Care Unit, Peking University Third Hospital, Beijing, China; ^3^ CAS Key Laboratory of Genomic and Precision Medicine, Beijing Institute of Genomics, Chinese Academy of Sciences, Beijing, China; ^4^ Institute of Materia Medica, Chinese Academy of Medical Sciences and Peking Union Medical College, Beijing, China; ^5^ Department of Laboratory Medicine, Tongji Hospital, Tongji Medical College, Huazhong University of Science and Technology, Wuhan, China; ^6^ Department of Medical Laboratory, the Central Hospital of Wuhan, Tongji Medical College, Huazhong University of Science and Technology, Wuhan, China; ^7^ Chinese Academy of Medical Sciences, Peking Union Medical College, Beijing, China; ^8^ China National Center for Bioinformation, Beijing, China; ^9^ University of Chinese Academy of Sciences, Beijing, China; ^10^ Department of Immunology, School of Basic Medical Sciences, Peking University, NHC Key Laboratory of Medical Immunology (Peking University), Beijing, China; ^11^ Department of Forensic Sciences, College of Criminal Justice, Naif Arab University of Security Sciences, Riyadh, Saudi Arabia

**Keywords:** coronavirus disease 2019, T cells, T cell receptor β repertoire, machine learning, immunology

## Abstract

**Background:**

The immune responses to severe acute respiratory syndrome coronavirus 2 (SARS-CoV-2) are crucial in maintaining a delicate balance between protective effects and harmful pathological reactions that drive the progression of coronavirus disease 2019 (COVID-19). T cells play a significant role in adaptive antiviral immune responses, making it valuable to investigate the heterogeneity and diversity of SARS-CoV-2-specific T cell responses in COVID-19 patients with varying disease severity.

**Methods:**

In this study, we employed high-throughput T cell receptor (TCR) β repertoire sequencing to analyze TCR profiles in the peripheral blood of 192 patients with COVID-19, including those with moderate, severe, or critical symptoms, and compared them with 81 healthy controls. We specifically focused on SARS-CoV-2-associated TCR clonotypes.

**Results:**

We observed a decrease in the diversity of TCR clonotypes in COVID-19 patients compared to healthy controls. However, the overall abundance of dominant clones increased with disease severity. Additionally, we identified significant differences in the genomic rearrangement of variable (V), joining (J), and VJ pairings between the patient groups. Furthermore, the SARS-CoV-2-associated TCRs we identified enabled accurate differentiation between COVID-19 patients and healthy controls (AUC > 0.98) and distinguished those with moderate symptoms from those with more severe forms of the disease (AUC > 0.8). These findings suggest that TCR repertoires can serve as informative biomarkers for monitoring COVID-19 progression.

**Conclusions:**

Our study provides valuable insights into TCR repertoire signatures that can be utilized to assess host immunity to COVID-19. These findings have important implications for the use of TCR β repertoires in monitoring disease development and indicating disease severity.

## Introduction

The ongoing COVID-19 pandemic, caused by severe acute respiratory syndrome coronavirus 2 (SARS-CoV-2), has had a profound global impact, resulting in unprecedented public health and socioeconomic consequences (1, 2). In 2020 and 2021 alone, COVID-19 was responsible for an estimated 14.9 million deaths worldwide (3). The clinical presentation of COVID-19 exhibits considerable variability, with the initial outbreak revealing a wide range of symptoms. While the majority of infected individuals experience mild to moderate symptoms or remain asymptomatic, approximately 10%–20% develop severe forms of the disease, such as acute respiratory distress syndrome, leading to increased mortality rates (4, 5). Understanding the pathogenesis of COVID-19 is of utmost importance to prevent disease progression and effectively manage the ongoing pandemic, especially in light of the resurgence of new cases globally and the potential for the pandemic to persist in the years ahead.

Immune responses are pivotal in host-pathogen interactions during infectious diseases, and numerous studies have highlighted their role in maintaining a delicate balance between protective effects and harmful pathological reactions in the context of SARS-CoV-2 and COVID-19 progression (6–11). T cells, also known as T lymphocytes, are integral components of the immune response against coronaviruses (12–14). Patients with more severe symptoms of COVID-19 often exhibit decreased levels of CD4+ and CD8+ cells, resulting in prolonged viral persistence and increased mortality rates compared to those with moderate symptoms (15, 16). T cells recognize pathogen-derived peptides by presenting them to the major histocompatibility complex (MHC) on virally infected cells through their hypervariable T cell receptors (TCRs). The TCRs are generated through the random recombination of variable (V), diversity (D), and joining (J) gene segments in the complementarity-determining region 3 (CDR3) of the receptor chain, a process known as V(D)J recombination (17). The beta (β) chain, formed by the connection of the D region with the V and J regions, is highly diverse and contains more information than the alpha chain (V and J), making it well-suited for characterizing T cell immune responses. Moreover, the β chain sequence is relatively short, enabling its analysis using high-throughput sequencing technologies (17). TCR repertoires exhibit dynamic composition and diversity, serving as important parameters in immune responses (18). While TCR repertoires are diverse and polyclonal under normal conditions, they can become biased during infection due to preferential selection of pathogen-specific TCR clones. Thus, real-time and quantitative tracking of T cell clones through TCR repertoire sequencing can provide insights into the expansion and contraction phases of antiviral defense and potentially aid in predicting clinical outcomes (17, 19). Given that SARS-CoV-2 is a novel coronavirus to which humans have not developed prior immune responses, investigating differences in TCR repertoires resulting from infection with this virus offers an innovative approach that may elucidate the mechanisms underlying antigen-specific immune pathology. While several studies have explored the characteristics of TCR repertoires in COVID-19 (20–24), comprehensive characterizations of TCR repertoires in a reasonably sized sample of infected individuals with varying severity levels and clinical follow-up remain scarce, and the relationship between TCR repertoire parameters and COVID-19 immunopathology remains unknown.

In this study, we conducted an in-depth analysis of the TCR β repertoires in the peripheral blood of 192 patients exhibiting moderate, severe, or critical symptoms of COVID-19, as well as 81 healthy controls. Through this analysis, we characterized TCR β repertoire signatures and identified specific TCR β clonotypes associated with SARS-CoV-2 infection. Additionally, we employed a machine learning algorithm to classify patients with COVID-19 of varying severities from the healthy controls.

## Materials and methods

### Sample collection and survival analysis

**Figure 1:**
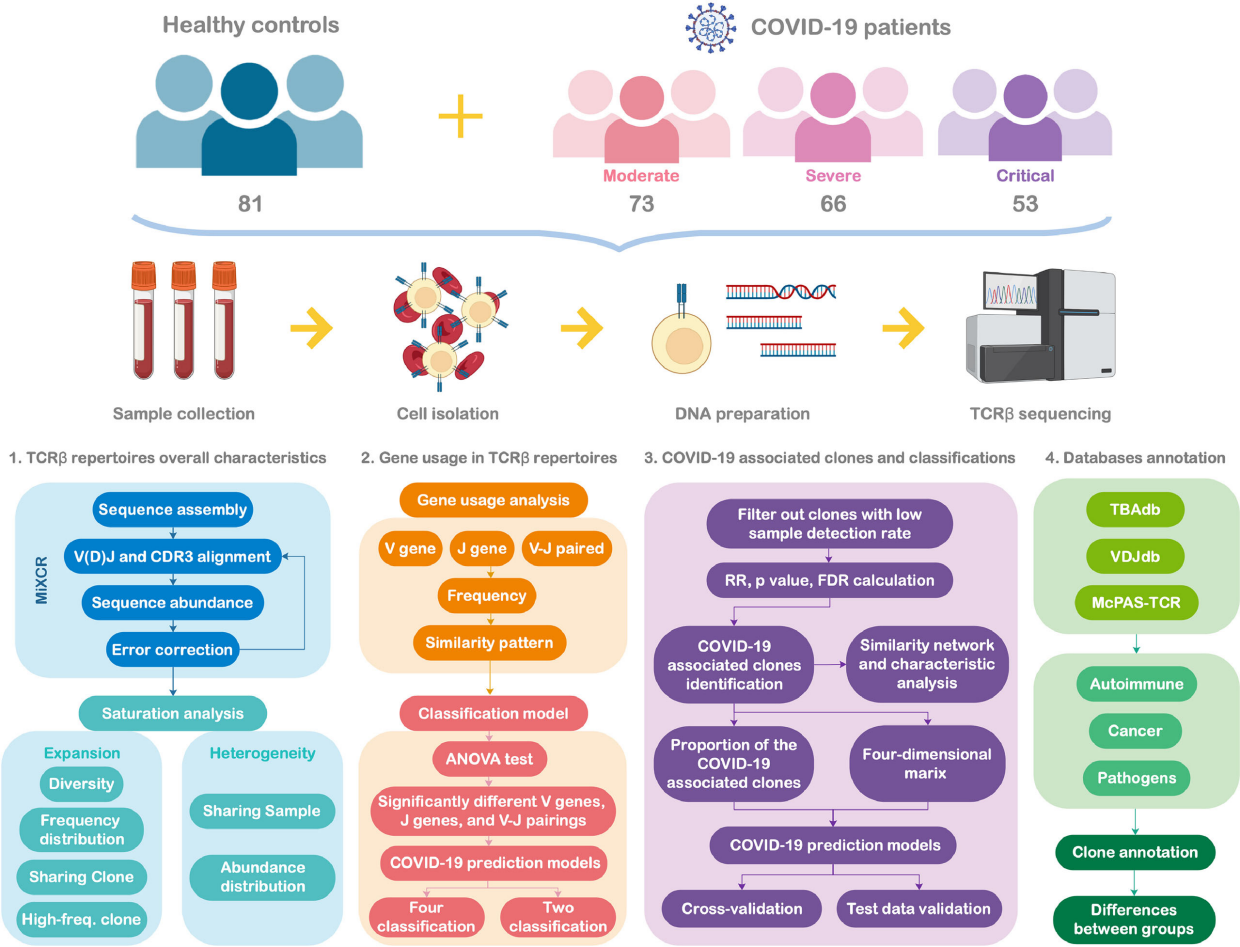
Study flowchart.

**Figure 1** presents the flowchart of the study design. This multicenter, prospective cohort study was conducted at three hospitals in Wuhan. Between March and April 2020, 192 patients aged at least 18 years and diagnosed with COVID-19 were consecutively enrolled. The patients were classified into three groups according to the *Protocol on Prevention and Control of Novel Coronavirus Pneumonia* (Edition 6, https://www.chinadaily.com.cn/pdf/2020/2.COVID-19.Prevention.and.Control.Protocol.V6.pdf. COVID-19. Prevention.and.Control.Protocol.V6.pdf) developed by the National Health Commission of the People’s Republic of China: moderate (fever, respiratory symptoms, etc., with abnormal findings on chest imaging), severe (respiratory rate ≥ 30/min, oxygen saturation ≤ 93% on room air at rest, arterial oxygen pressure/fraction of inspiration oxygen ≤ 300 mmHg, or progressive clinical symptoms and lung imaging showing > 50% significant progression of the lesion within 24–48 h), and critical (respiratory failure requiring mechanical ventilation or shock combined with other organ failure and requiring intensive care unit supervision). Each patient was prospectively followed-up till hospital discharge or death. The clinical outcomes of patients with COVID-19 were evaluated after 28 days of follow-up. All the patients were classified into three types, and the severity of disease classification was used for comparing the state of the disease on the 1^st^ (baseline) and 28^th^ day of follow-up.

Type 1: Deteriorate: Patients progressed from moderate to severe/critical COVID-19 or from severe to critical COVID-19, along with those who died owing to any reason during the 28-day follow-up.

Type 2: Unchanged: Disease severity remained the same as that at baseline.

Type 3: Improved: Patients with alleviated symptoms or those who recovered from COVID-19, with at least one of the following criteria being achieved: 1) body temperature was back to normal for > 3 days; 2) respiratory symptoms improved, i.e., successfully weaning off mechanical ventilation; 3) pulmonary imaging showed absorption of inflammation; and 4) nucleic acid tests of respiratory tract samples were negative two consecutive times, with a sampling interval at least 24 h.

The Ethical Review Board of Peking University Third Hospital approved the study protocol in March 2020, with the exemption of informed consent from participants (IRB00006761-M2020083). We also recruited 81 HCs from a previous cohort study that was conducted by Fuwai Hospital, China National Center for Cardiovascular Diseases, from March 2018 to December 2019, i.e., before the COVID-19 pandemic.

Patients with complete clinical data and survival information (n = 167) were included in the survival analysis. Kaplan–Meier survival curves and Cox proportional hazards models were established using the survival (version 3.2–11, https://CRAN.R-project.org/package=survival) and survminer (version 0.4.9) packages in R software (25). Schoenfeld residuals were used to assume proportional hazards before Cox analysis. The log-rank test was used to assess statistical significance. A P-value of < 0.05 was considered statistically significant.

### High-throughput TCR β repertoire sequencing

Peripheral blood samples were collected from 273 subjects. The DNA was extracted utilizing QIAamp DNA Blood Mini Kit (NO. 51306, Qiagen, Hilgen, Germany), and measured using NanoDrop 2000 spectrophotometer (Thermo Fisher Scientific, Waltham, MA, USA) to determine concentration. The DNA served as the template for PCR ampliflication, originating the library of TCR β. The step 1 PCR ampliflication protocol was as follows: 95°C for 5 min; 95°C for 30 s, 59°C for 30 s; 72°C for 1 min for 30 cycles; and 72°C for 10 min. The step 2 PCR ampliflcation protocol was as follows: 98°C for 2 min; 98°C for 30 s, 65°C for 30 s; 72°C for 30 s for 10 cycles; and 72°C for 5 min. The PCR products were purified, and the barcodes were confirmed. The CDR3 gene fragments of the TCR β chain were amplified through a multiplex PCR amplification reaction. Then, they were sequenced using the Illumina HiSeq2000 platform from the genomic DNA. The CDR3 nucleic acid sequences of the TCR β chain were aligned based on the definition established by the International ImMunoGeneTics (IMGT) collaboration (26). An algorithm was utilized to identify the V, D, and J segments that contributed to the CDR3 region based on established protocol (26). Only productive reads were evaluated for downstream analyses since sequences with frameshifts or stop codons cannot produce functional proteins.

### Preprocessing of raw amplicon PCR sequencing data and alignment to the IMGT database

To decrease the effects of sequencing errors, we first used Cutadapt software (version 2.10) (27) to strictly filter the raw sequencing data based on four criteria and delete the following sequences (1): sequences containing linker sequence contamination (2); those with > 5% sequences of unknown bases (N) (the threshold of N base ratio was set to < 5%) (3); those with an average quality score of < 20 (based on the Illumina 0–41 quality system); and (4) those with low-quality bases (Q score < 20) or very short sequences (< 60 bp in length).

The human TRBV sequence was downloaded from the IMGT database (https://www.imgt.org/vquest/refseqh.html), and the reference sequence was uploaded to GitHub (https://github.com/Na-Yuan-BIG/TCR-rep/tree/main/imgt). After obtaining high-quality sequencing data by preprocessing the raw data, we used MiXCR software (version 3.0.13) (28) to complete TCR clonotype identification of the sequenced fragments, which can realize the conversion from raw sequence to quantitative clonotype data. We used the analyze mode to completely align, assemble, and perform assembleContigs and exportClones analysis of each sample in one command line (mixcr analyze amplicon–species hsa–starting-material DNA–5-end v-primers–3-end j-primers–adapters adapters-present–receptor-type TRB). The 192 samples used in the analysis had a mapping rate of > 70%. Then, we used the convert function of Vdjtools (version 1.2.1) (29) to convert the output of MiXCR into a text file in VDJtools format for subsequent analysis.

### Overall characteristics of the TCR β repertoires of COVID-19 and HC samples

All analyses were performed using R (version 3.6.3, https://www.R-project.org), the immunarch (version 0.6.5) package, and Python (version 3.8.3).

### Gene usage analysis and COVID-19 prediction

Comparative analyses of VJ gene usage were performed by clustering information based on its occurrence frequency in each sample. Similarities in usage patterns between different groups were determined using Spearman’s correlation. To identify the significantly different V genes, J genes, and VJ pairings between different groups, one-way analysis of variance (ANOVA) and post-hoc multiple comparisons were separately performed on the frequency of V gene/J gene/VJ pairing. The classification performance of the three different models of naive Bayes classifier, multiple logistic regression model, and random forest model was compared using V gene/J gene/VJ paring separately; we observed that the random forest model has better classification performance. Therefore, we obtained four classification results (control/moderate/severe/critical) by separately using V gene/J gene/VJ paring based on the random forest model. To ensure model reliability, we used the same method to build a random forest classification model in the training set and verified the classification performance of the model in the test set. To classify any two groups among the four sample groups, we also established a random forest classification model using the above method.

### COVID-19-associated TCR β identification

In each sample, we defined clones with the same V gene, J gene, and CDR3 amino acid sequences as the same clone. The detected clones and their abundance information in all samples were integrated to obtain the frequency abundance of each TCR clone in each sample. During integration, the clones with no more than 10 detection samples were filtered to improve subsequent calculation efficiency.

The detection rate of each clone in the HC and COVID-19 groups was calculated (i.e., the number of samples detected in the clone) and recorded as cC and cD, and the nondetection rate in the corresponding groups were calculated and recorded as cnC and cnD, respectively. The relative risk (RR) index of the clones was calculated as follows:


$$RR=\frac{\frac{cD}{cD+cnD}}{\frac{cC}{cC+cnC}}$$

When RR is > 1, it indicates that the clone has a higher detection rate in the COVID-19 group, which is positively correlated with an infection status of COVID-19. Based on the detection rate of the clones in the HC and COVID-19 groups, Fisher’s exact test was performed to evaluate the differences in the distribution of the clone detection rate between the two groups, and P-values of descriptive statistical significance were obtained. Then, the corrected p-values (FDR) were determined using p.adjust (method = “fdr”).

Finally, the RR and FDR values were combined to identify the COVID-19-related clones and protective clones. Here, we used three filters:

(1) Large panel: 284 COVID-19-specific clones were identified under the threshold of RR > 1 and FDR < 1e−5. Simultaneously, under the threshold of RR < 1 and FDR < 1e−25, 276 clones were identified in the HC group, which were called protective clones.
(2) Middle panel: 102 COVID-19-specific clones were identified under the threshold of RR > 1 and FDR < 1e-6. Simultaneously, under the threshold of RR < 1 and FDR < 5e−27, 101 clones were identified in the HC group, which were called protective clones.
(3) Mini panel: Under stricter threshold conditions of RR > 1 and FDR < 1e−8, 12 COVID-19-specific clones that were significantly different from those of the HC group were identified in the COVID-19 group.

### Characteristic analysis of COVID-19-associated TCR β clones

Based on the results presented in the “COVID-19-associated TCR β identification” section, the characteristics of COVID-19-specific, protective, and random clones were studied.

The same number of COVID-19-specific clones and non-COVID-19-related clones, as random clones, were randomly sampled, followed by the evaluation of clone abundance, CDR3 amino acid sequence length, and CDR3 amino acid sequence similarity among the COVID-19-specific, protective, and random clones. For comparison, 100 random sampling experiments were performed to avoid sampling bias caused by one-time random sampling. Minimum Edit Distance was used to compare the similarity between the CDR3 amino acid sequences (30).

### Similarity network analysis of differential clones and high-frequency clones

The differential clones between the COVID-19 and HC groups were screened (FDR < 0.001). The CDR3 sequences of these clones were separately extracted for each sample group using GLIPH2 (Version: 0.01) (31) software, which performs clustering. For clustering results, single clones were filtered without clustering and the network diagram was constructed using the R package igraph (1.2.8) (32). Similarly, for high-frequency clones, 1000 clones (detected in > 10 samples) with the highest frequency in the COVID-19 and HC groups, respectively, were extracted after gliph2 clustering; the similarity network was constructed using Gephi2 (0.9.2) (33).

### Construction of the COVID-19 prediction models using COVID-19-associated TCR β clones

We used two methods to construct the COVID-19 prediction model.

Route 1: The proportion of the COVID-19-specific clones was calculated in all samples based on the COVID-19-specific clones (102 clones) obtained in the “COVID-19-associated TCR β identification” section to the sample’s own unique TCR β clones. All samples were classified and predicted according to this index.

Route 2: Dimensionality reduction was used to generate a four-dimensional feature value (34) for each sample using the distribution information of the COVID-19-specific clones in different sample groups. Then, the four-dimensional feature values were calculated under several different FDR thresholds (calculated according to the “COVID-19-associated TCR β identification” section), and the random forest classification model was constructed in the training set. The performance of the classification model was verified in the test set. The four-dimensional features used were F_uinq_/F_abund_/N_d_/N_C_.

F_uinq_: Number of unique COVID-19-specific clones in the sample accounts for the proportion of all unique TCR βs in this sample.
F_abund_: Number of abundant COVID-19-specific clones in the sample accounts for the proportion of all unique TCR βs in this sample.
N_d_: Cumulative number of COVID-19-specific clones contained in the samples detected in the COVID-19 group.
N_C_: Cumulative number of COVID-19-specific clones contained in the samples detected in the HC group.

### TCR-specific databases annotation

Human TRB data were downloaded and integrated from the TCR-specific databases TBAdb (35), VDJdb (36), and McPAS-TCR (37). All clones were divided into three categories: Autoimmune, Cancer, and Pathogens, corresponding to 9, 10, and 22 subcategories, respectively. Based on the annotation information of these three databases, disease-related information in the databases was obtained using the V gene, J gene, and CDR3 sequence for pattern matching.

The percentage of clones present in the databases was calculated using the total number of unique clones for all sample groups, and the distribution of the relevant clones in the three major categories of the 41 disease subcategories was compared in each sample group. The Mann–Whitney test was performed in the HC and COVID-19 groups. In addition, the clones identified in the samples in the HC and COVID-19 groups were compared with the COVID-19-specific TRB clones in the three databases.

## Results

### Sample characteristics

**Table 1** presents an overview of the characteristics of the 192 patients with COVID-19 and the 81 healthy controls (HCs). The samples were collected from COVID-19 patients before the availability of COVID-19 vaccines or immunological agents. Patients received supportive treatments, including mechanical ventilation, following the 6th tentative protocol, which is well-documented and publicly available. The analysis revealed a significant association (P < 0.001) between aging and an increased risk of more severe symptoms (P = 5.91e−09). Patients with more severe symptoms had a significantly higher prevalence of hypertension. Interleukin (IL)-6 serum levels demonstrated a strong and significant association (P = 3.19e−06) with disease severity, while the levels of other ILs did not reach significance, even at the nominal level. Survival analysis confirmed the highly significant impact of IL-6 (P = 8.56e−23) on the survival of COVID-19 patients and also identified a nominally significant effect of IL-2R on survival (P = 0.0003, **Figure S1**, **Table S1**). Other laboratory test indicators, including WBC, HGB, LYMPH, NEUT, TCHOL, ALB, LDH, and CRP, showed significant associations. Overall, these characteristics were consistent with those observed in previous clinical and epidemiological studies (38–42).

**Table 1:** Clinical characteristics of the study cohort.

Indexes	Control (n = 81)	Moderate (n = 73)	Severe (n = 66)	Critical (n = 53)	*P*-value
Age	50.5 (44.0–57.0)	63.0 (45.0–71.0)	66.5 (58.2–78.8)	66.5 (55.8–73.0)	***,#
Gender(F/M)	29/42	40/33	37/29	13/27	$,+
Past medical history					
Hypertension, n(%)	–	6 (8.2)	28 (42.4)	14 (58.3)	###,+++
Diabetes, n(%)	–	6 (8.2)	10 (15.2)	8 (33.3)	ns
Smoking					
Never smoker, n(%)	–	2 (66.7)	55 (87.3)	15 (65.2)	ns
Former smoker, n(%)	–	1 (33.3)	5 (7.9)	2 (8.7)	
Current smoker, n(%)	–	0 (0.0)	3 (4.8)	6 (26.1)	
CBC					
WBC (10^9/L)	5.5 (4.6–6.5)	5.8 (4.8–6.9)	6.2 (4.4–7.7)	9.8 (7.5–16.0)	**,$$$,+++
HGB (g/L)	148.0 (138.8–154.8)	128.0 (114.0–140.0)	117.5 (103.0–127.0)	105.5 (88.0–129.0)	***,#,++,
LYMPH#	–	1.2 (0.8–1.6)	0.8 (0.6–1.2)	0.9 (0.6–1.3)	###,
NEUT%	57.0 (51.0–62.8)	68.7 (59.5–76.3)	76.1 (66.3–85.9)	85.5 (69.7–90.2)	***,##,+++
TCHOL(mmol/L)	4.7 (4.3- 5.4)	4.1 (3.4- 4.9)	3.8 (3.2- 4.4)	3.3 (2.9- 4.1)	***,+
Hepatorenal					
ALB (g/L)	–	41.1 (37.7–44.4)	34.2 (32.3–39.1)	34.1 (29.9–38.5)	###,+++
Tbil (µmol/L)	–	12.2 (8.8–16.4)	9.8 (7.2–15.0)	13.5 (8.3–19.9)	*ns*
ALT (U/L)	–	15.0 (14.0–24.0)	24.0 (15.0–32.5)	27.5 (21.8–50.2)	*ns*
CREA (µmol/L)	81.0 (71.4–92.4)	72.2 (59.8–88.3)	70.5 (59.2–103.8)	64.0 (52.8–86.5)	**
LDH (U/L)	168.5 (149.8–196.8)	181.0 (150.0–213.0)	263.0 (196.8–337.0)	427.0 (298.0–481.0)	***,###,$$,+++
CRP (mg/L)	1.2 (0.5–2.1)	0.4 (0.1–2.1)	19.5 (2.8–72.0)	63.6 (38.1–86.0)	***,###,$$,+++
Interleukin					
IL-1β (pg/mL)	–	–	5.0 (5.0–8.1)	5.0 (5.0–7.5)	*ns*
IL-2R (U/mL)	–	–	811.0 (514.5–1093.0)	863.0 (496.0–958.0)	*ns*
IL-6 (pg/mL)	–	3.1 (1.7–8.1)	13.9 (4.6–33.1)	41.5 (16.4–82.3)	###,$,+++
IL-8 (pg/mL)	–	–	12.7 (8.1–20.0)	12.5 (12.5–35.6)	*ns*
IL-10 (pg/mL)	–	–	5.9 (5.0–8.7)	6.7 (5.9–7.1)	*ns*
T cell subsets					
LY%	–	72.7 (66.8–77.6)	76.0 (68.6–78.6)	77.1 (69.9–77.4)	*ns*
CD3Abs (cells/ul)	–	1704.0 (1149.5–2167.5)	650.0 (344.8–909.8)	431.0 (190.5–679.0)	*ns*
Th%	–	41.6 ± 10.4	48.3 ± 12.6	43.8 ± 10.4	*ns*
Th# (cells/ul)	–	670.0 (436.5–1339.5)	388.5 (217.2–589.0)	238.0 (105.0–356.0)	*ns*
Ts%		26.0 (20.1–32.5)	20.3 (17.3–21.5)	24.6 (24.4–25.2)	#
Ts# (cells/ul)	–	629.0 (460.0–792.5)	230.0 (81.0–307.8)	188.0 (93.0–326.5)	*ns*
Th/Ts	–	0.7 (0.7–1.9)	1.7 (1.3–2.7)	1.3 (1.1–1.8)	*ns*
Mechanical Ventilation					
Mechanical Ventilation (Hours)	–	0.0 (0.0–0.0)	0.0 (0.0–0.0)	240.0 (0.0–522.0)	##,$$$,+++
LOS					
LOS (days)	–	25.0 (21.0–31.0)	27.5 (21.0–37.8)	25.0 (19.2–43.2)	*ns*
ICU (days)	–	0.0 (0.0–0.0)	0.0 (0.0–0.0)	17.5 (3.8–25.2)	##,$$$,+++
Clinical outcome					
Recovered, n(%)	–	72 (98.6)	57 (86.4)	17 (70.8)	#,$,+++
Survival, n(%)	–	1 (1.4)	5 (7.6)	4 (16.7)	
Death, n(%)	–	0 (0)	4 (6.1)	3 (12.5)	

a. n(%), median(p25-p75), mean ± SD.

b. One-way analysis of variance (ANOVA) was employed to calculate the P values. Statistically significant difference between the groups (Cotrol-COVID-19: *, Moderate-Severe: #, Severe-Critical: $, Moderate-Critical: +) as *** P ≤ 0.001, **P ≤ 0.01, *P ≤ 0.05 and ns P > 0.05.

c. LY%: CD3+CD19; CD3Abs: CD3+CD19-; Th%: CD3+CD4+; Ts%: CD3+CD8+; Th/Ts: CD4/CD8.

d. for CREA p-vlaue ** shows that some patients have already used renal replacement therapy during the blood collection period, and creatinine cannot fully reflect renal function.

e. Normal range:

for CBC indicators, WBC: [3.5–9.5], HGB: [130–175], LYMPH#: [1.1–3.2], NEUT%: [40–75], TCHOL: [2.9–5.7].

for Hepatorenal indicators, ALB: [35–52], Tbil: ≤26, ALT: ≤41, CREA: [59–104], LDH: [135–225], CRP: < 1.

for Interleukin indicators, IL-1β: < 5, IL-2R: [223–710], IL-6: < 7, IL-8: < 62, IL-10: < 9.1.

for T cell subsets indicators, LY%: [50–84], CD3Abs: [955–2860], Th%: [27–51], Th#: [550–1440], Ts%: [15–44], Ts#: [320–1250], Th/Ts: [0.71–2.78].ns = no significance.

### Decreased diversity of TCR β repertoires in patients with COVID-19

**Figure 2:**
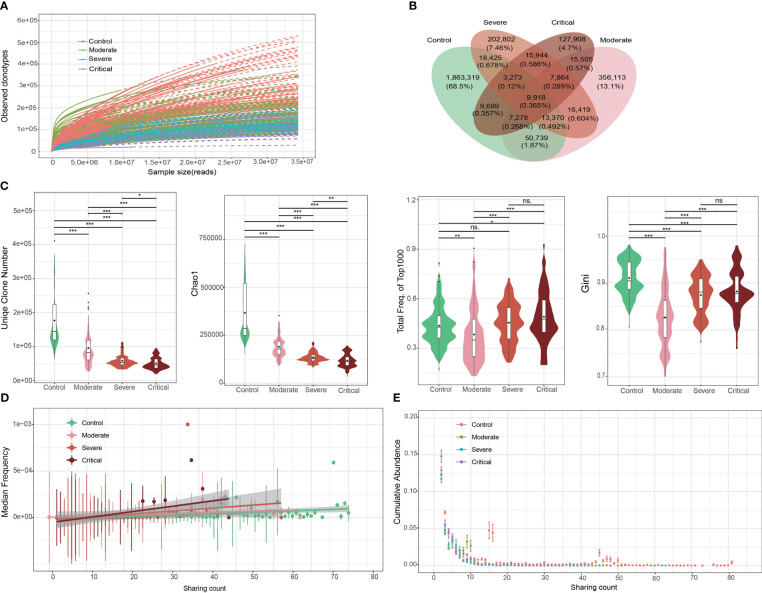
Overall characteristics of the T cell receptor (TCR) β repertoires in patients with COVID-19 and healthy controls (HCs). **(A)** Saturation analysis of each group. Sequencing data were randomly selected to determine the number of clonotypes detected and extrapolated to the size of the largest samples. Solid and dashed lines represent the interpolated and extrapolated regions of the rarefaction curves, respectively, and points indicate the exact sample size and diversity. **(B)** Overlap between each group of public clones. **(C)** Obvious clonal expansion from the aspects of TCR β repertoire diversity in COVID-19 development. Horizontal line is the median, and dot is the average value in the boxplot. **(D)** Trend of changes in the average abundance of clones under different detection sample numbers for each group. **(E)** Trend of changes between the total abundance of clones under different detection sample numbers for each group. ***P ≤ 0.001, **P ≤ 0.01, *P ≤ 0.05. ns = no significance.

Following the isolation of T cells and high-throughput TCR β repertoire sequencing, we obtained an average of 1.2e7 to 1.7e7 pair-end sequences per sample (**Table S2**). However, due to sample heterogeneity and the presence of numerous low-abundance clones, capturing the full range of clonotypes in a single sample was challenging. Even when sequencing the maximum number of samples, most samples approached saturation, as depicted in **Figure 2A**, where the number of observed clones continued to increase until reaching 3.5e7 reads. Nevertheless, our sequencing data revealed a significant decrease in the number of clones as COVID-19 severity increased (**Figure 2A**; **Table S2**).

To investigate clonal expansion in individuals with COVID-19, we assessed the diversity of TCR β repertoires using various parameters, including the number of clonotypes, Chao1 index, accumulative frequency, Gini index, and clonal frequency. In patients with COVID-19, the top 1000 TCR clones exhibited a reduced number of clonotypes and Chao1 index, along with increased accumulative frequencies (**Figure 2C**). This finding suggests that clonal diversity decreased as a subset of SARS-CoV-2-specific clones expanded, indicating a more active state of the immune response following viral infection. Similar patterns were observed in the accumulative frequency analysis focusing on the top 100 TCR clones, although to a lesser extent than with the top 1000 TCR clones. Notably, the magnitude of clonal expansion within the top 1000 frequent clones was greater than observed in a previous study on systemic lupus erythematosus (SLE) and RA (34), where clonal expansion was evident within the top 100 frequent clones. This suggests that a broader range of TCR clones contributes to SARS-CoV-2-specific immune responses compared to SLE and RA.

The Gini index, which measures clonotype uniformity, was significantly decreased in patients with COVID-19 compared to healthy controls (**Figure 2C**). This finding was unexpected since the rapid expansion of a small number of clones, disrupting the uniformity of the original clone distribution, would typically lead to an increased Gini index. However, our observations align with those in SLE and RA patients (34), where the Gini index was also significantly decreased and then increased with disease severity. The decrease in the Gini index in COVID-19 patients suggests clonal expansion following infection, albeit in an unexpected direction.

Clonal frequency, a common measure of clonal amplification, demonstrated that public clones (shared between two or more samples) in patients with severe and/or critical disease had significantly higher frequencies compared to those in patients with moderate disease and/or healthy controls (**Figures 2D, E**). This finding supports the hypothesis that patients with severe and critical disease exhibit a more robust immune response and greater clonal expansion.

### Heterogeneity of the TCR β repertoire in patients with COVID-19

The TCR β repertoire is well-known for its high level of heterogeneity, wherein individuals typically share only a small number of clones. In our study, we confirmed this characteristic (**Figure 2B**). More specifically, over 90% of clones were found in only one individual, and the shared clonotypes between any two individuals accounted for only 0.23%–4.22% of the total clonotypes. Importantly, we observed a significantly increased heterogeneity in patients with COVID-19 compared to healthy controls, as evident in the clonotype distribution (**Figure S2**). The likelihood of finding shared clones between any pair of healthy controls was much higher than that between any pair of patients. Notably, the heterogeneity did not significantly differ among patients with varying levels of disease severity. Remarkably, the observed increase in heterogeneity in patients with COVID-19 was consistent with the decrease in the Gini index.

### Classification of the patients using V and J genes

**Figure 3:**
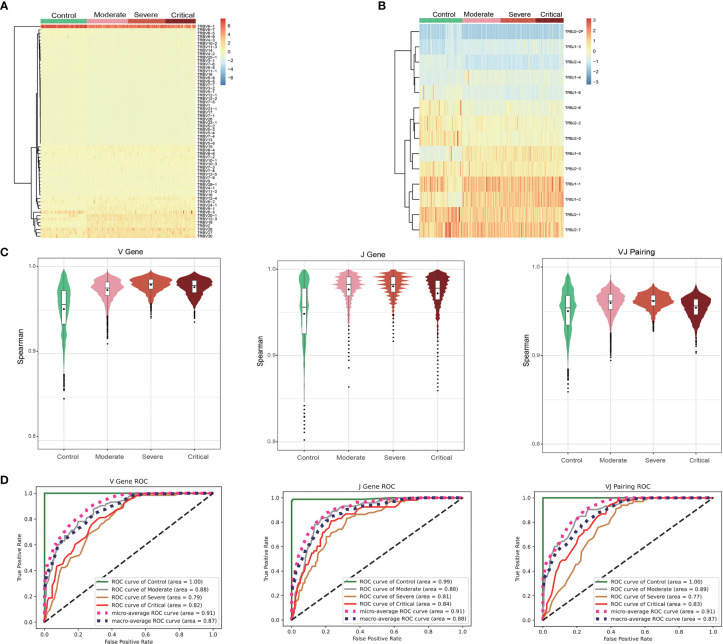
Gene usage analysis of the T cell receptor (TCR) β repertoires. Frequency of V gene **(A)** and J gene **(B)** usage in different samples. **(C)** Frequency of the similar patterns of V gene, J gene, and VJ pairing in different groups. **(D)** ROC curves showing the performance of the classification into four groups using the V gene, J gene, and VJ pairing *via* 10-fold cross-validation.

Cluster analysis demonstrated that patients could be effectively differentiated from healthy controls based on the usage of V genes (**Figure 3A**) or J genes (**Figure 3B**), while the differentiation among patients with different disease severity levels was less pronounced. Overall, the usage of V genes, J genes, and VJ pairings showed similarities between individuals (V: r > 0.85, J: r > 0.77, and VJ: r > 0.86; **Figure 3C**). However, gene usage within patients exhibited significantly higher similarity compared to that within healthy controls (V: r > 0.91, J: r > 0.86, VJ: r > 0.89; **Figure 3C**). Through one-way ANOVA analysis of gene usage, we identified 46 out of 63 V genes, 11 out of 14 J genes, and 536 out of 772 VJ pairings that displayed significant differences between the patient group and healthy controls (P < 0.001, **Table S3**). Subsequently, prediction modeling was performed using the significant genes obtained from ANOVA, employing three commonly used methods: naïve Bayes, multiple logistic regression, and random forest. Ten-fold cross-validations demonstrated that the random forest classifier achieved the highest accuracy (**Figure S3**), with a microaverage AUC of 0.91 and a macroaverage AUC of 0.87–0.88 for classifying all samples into four groups (Control, Moderate, Severe, and Critical; **Figure 3D**). Examining each group individually, the Control group was almost perfectly classified using the V gene, J gene, and VJ pairings (AUC > 0.99). The accuracies for classifying the Moderate group (V: AUC = 0.88, J: AUC = 0.88, and VJ: AUC = 0.89) and the Critical group (V: AUC = 0.82, J: AUC = 0.84, VJ: AUC = 0.83) were slightly lower but still satisfactory. In contrast, the accuracy for classifying the Severe group was the lowest (V: AUC = 0.79, J: AUC = 0.81, VJ: AUC = 0.77).

### Identification and characteristics analysis of COVID-19-associated TCR β clones

**Figure 4:**
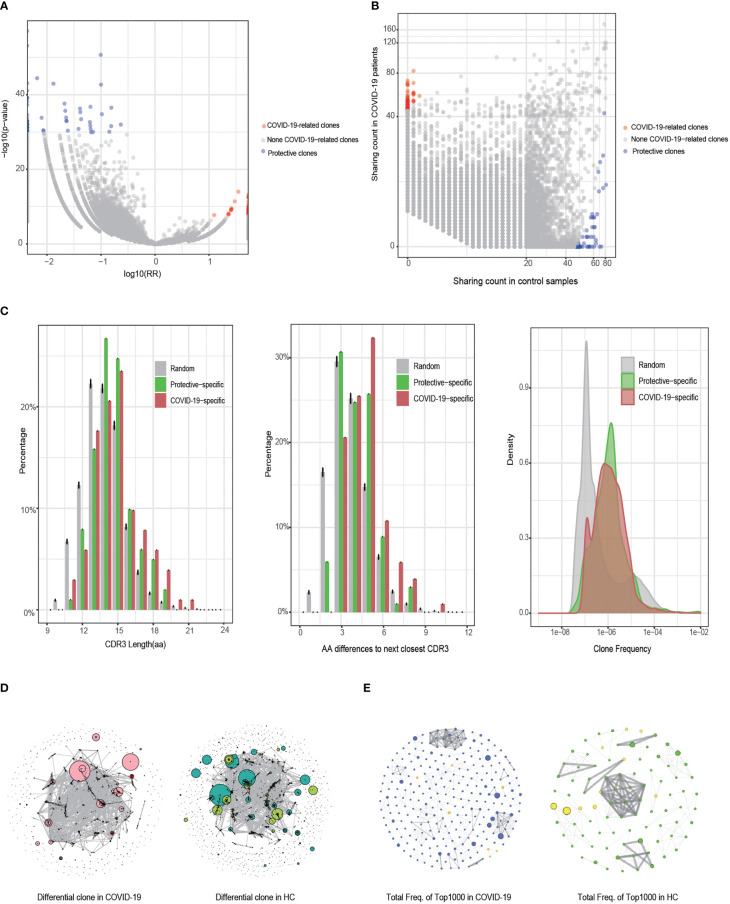
Identification and characteristic analysis of COVID-19-associated T cell receptor (TCR) β clones. **(A)** Differences in the detected clones between the COVID-19 and healthy control (HC) groups (Fisher’s exact test). The horizontal axis is the clone’s relative risk (RR) index and the vertical axis is the FDR value after correction using Fisher’s exact test. **(B)** Distribution of the clone detection sample numbers between the COVID-19 and HC groups. **(C)** Comparison of COVID-19-specific, protective, and random clones in terms of CDR3 length, CDR3 sequence similarity, and clone abundance. **(D)** Similarity network of the CDR3 sequences of the clones with a difference in FDR values of < 0.001 between the COVID-19 and HC groups. Clones identified as dark red dots are COVID-19-specific clones, whereas those identified as light green dots are protective clones. The size of the dot represents the clone’s cumulative frequency in all samples. **(E)** Similarity network of CDR3 sequences in the 1000 clones (dominant clones) with the highest frequency in the COVID-19 and HC groups. The node degree of the cluster graph in **(D, E)** is > 2.

Analysis of COVID-19-associated TCR β clones identified a total of 36,597 differential clones that were specific to COVID-19 under an FDR threshold of < 0.001. Using a more stringent threshold of 1e−6, we identified 102 risk clones associated with a significantly increased risk of COVID-19 (**Figure 4A**). All these risk clones exhibited a RR greater than 10.8. Additionally, applying the same threshold (1e−6) for detecting protective clones, we identified 12,892 clones. To match the number of protective clones to the risk clones, we employed a highly stringent FDR threshold of ≤5e−27, resulting in the identification of 101 protective clones, all with an RR of less than 0.3 (**Figure 4A**). Notably, the 102 risk clones showed high detection rates in patients with COVID-19 (greater than 24%, up to 44%), while the 101 protective clones exhibited high detection rates in healthy controls (greater than 58%, up to 100%, **Figure 4B**).

Comparing the 102 COVID-19-specific risk clones, 101 protective clones, and 100 randomly resampled clones (repeated for 1000 replicates), we found that both risk and protective clones had higher frequencies than random clones (**Figure 4C**, right panel). Furthermore, the risk clones had significantly longer CDR3 amino acid sequences compared to random and protective clones, indicating an enrichment of longer CDR3 sequences (> 16 amino acids) in the risk clones (**Figure 4C**, left panel). The risk clones also exhibited a significantly longer smallest edit distance of CD3 sequences compared to the random and protective clones, with an enrichment of the smallest edit distances of ≥5 in the risk clones (**Figure 4C**, middle panel).

We performed GLPH2 clustering analysis of the CDR3 sequences to assess the similarity among the 36,597 differential clones (FDR < 0.001) in the COVID-19 and healthy control groups (**Figure 4D**). As expected, the differential clones showed a higher degree of connectivity and a smaller number of clusters in patients with COVID-19 compared to healthy controls, indicating a higher similarity in CDR3 sequences among COVID-19 patients. To further investigate this, we focused on clones shared by at least ten individuals and among the 1000 most frequent clones and repeated the GLPH2 clustering analysis (**Figure 4E**). This analysis revealed a greater number of connective nodes (n = 261) in patients with COVID-19 compared to healthy controls (n = 89), providing additional evidence for the higher similarity among these clones in COVID-19 patients. In summary, compared to random and protective clones, COVID-19-specific risk clones tended to utilize longer CDR3 sequences and displayed increased similarity, likely adapting to the complex antigen exposure environments encountered in the disease.

### Construction of COVID-19 prediction models using COVID-19-associated TCR β clones

**Figure 5:**
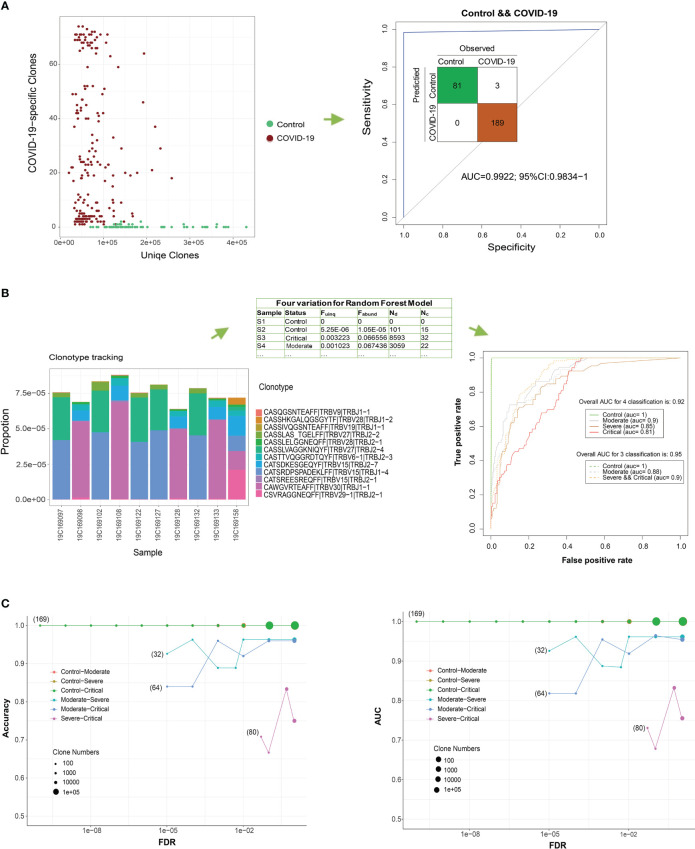
COVID-19 detection using T cell receptor (TCR) β clones from different classification models. **(A)** Distribution of the number of COVID-19-related clones in all the unique TCR βs of the sample. ROC curve showing the performance of all COVID-19 samples according to this ratio. **(B)** Frequency distribution bar plot of the 12 most significant COVID-19-related clones (FDR < 1e−8). ROC curve showing the performance of the test COVID-19 samples according to the random forest model using four variables. **(C)** Classification performance evaluations of the accuracy and AUC of the test set for two groups under different FDR thresholds.

The classification of patients with COVID-19 and healthy controls based on the ratio of COVID-19-specific risk clones to all unique TCR β clones (Fuinq > 1.5e−05, **Figure 5A**) yielded accurate results. Using the pool of 102 risk clones identified in our study, the classification achieved an AUC of > 0.99, and expanding the number of risk clones to 284 resulted in perfect classification (AUC = 1.00).

In a more stringent approach, we narrowed down the pool of clones to the top 12 COVID-19-specific clones based on a stricter threshold (FDR < 7.5e−9 and RR > 29.9, **Figure 5B**). Using the four parameters (Fuinq, Fabund, Nd, and NC) of these top 12 associated clones as predictors, a random forest classifier exhibited reasonably accurate classification results in differentiating patients from controls during 10-fold cross-validation (overall AUC for the four-parameter classification was 0.92; 95% CI: 0.905−0.939). Moreover, the classifier satisfactorily distinguished severe and critical patients from controls, achieving an AUC of 0.91 (overall AUC for three-parameter classification was 0.95; 95% CI: 0.940−0.965). To mitigate overfitting, we further divided our sample into 80% for training and 20% for testing. We replicated the marker selection (12 clones) and model building analyses on the training set and evaluated the predictive performance on the test set (**Figure 5C**). Generally, increasing the number of clones in the random forest model resulted in improved accuracy, with the lowest performance observed when differentiating critical from severe forms of the disease.

### Clinical implication of TCR β clones

We examined the correlation between IL levels and the abundance of COVID-19-specific clones in a subset of our patients (n = 32) with available IL data. While IL-6 and IL-2R were significantly associated with patient survival, they did not show a significant correlation with COVID-19-specific risk clones (P > 0.05). However, in our study, we found a significant positive correlation between IL-1 and the abundance of COVID-19-specific risk clones (*r* = 0.57, P = 7e-4, **Figure S4**). This finding suggests a potential connection between ILs and TCR β repertoires in patients with COVID-19. Further investigations with larger sample sizes are warranted to explore and confirm these correlations.

### Annotation of clonotypes using immune receptor databases

**Figure 6:**
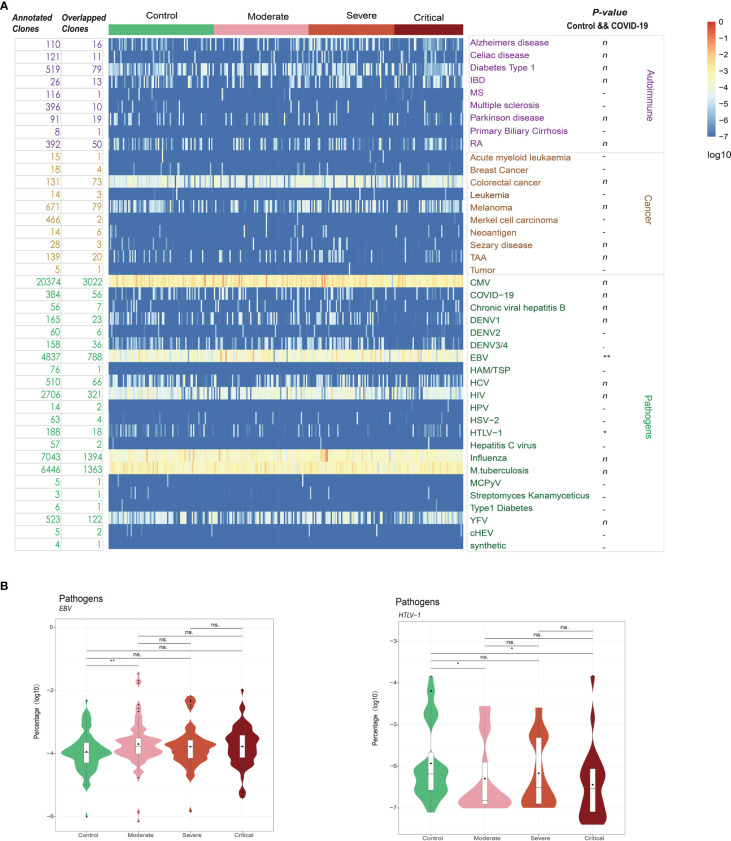
**(A)** Database annotation of the T cell receptor (TCR) β repertoire for each sample using TBAdb, VDJdb, and McPAS-TCR. **(B)** The percentage of annotated specific clones for significantly different groups. **P ≤ 0.01, *P ≤ 0.05 and ns P > 0.05. ns = no significance.

To explore potential connections between COVID-19 and other diseases, we utilized immune receptor databases (TBAdb, VDJdb, and McPAS-TCR) to annotate clonotypes. A total of 47,874 human TRB clones were integrated and categorized into Autoimmune, Cancer, and Pathogens, with further subcategories. Among these clones, 7,630 overlapped with our data, and 384 were annotated as COVID-19-specific clones, with 56 of them overlapping with our data. However, the distribution of these 56 clones did not exhibit significant differences among the four groups (HCs and patients with moderate, severe, and critical COVID-19; ANOVA FDR = 0.45, **Figure 6A**; **Figure S5**). The limited overlap between the COVID-19-specific clones annotated in published datasets and those identified in our study may be attributed to the high heterogeneity of the TCR β repertoire in patients with COVID-19 and the incompleteness of public TCR databases. Interestingly, we observed a significant difference between patients with COVID-19 and HCs in relation to Epstein-Barr virus (EBV; FDR = 0.007, **Figure 6A**). In publicly available datasets, 5,679 clones were annotated as EBV-specific clones, with 788 of them overlapping with our data. These EBV-specific clones showed a significant trend of increased frequency with higher severity levels of COVID-19 (**Figure 6B**). Another noteworthy finding was the significant association with human T lymphocytic leukemia virus type I (HTLV-1 virus; FDR = 0.016). Similarly, the distribution frequency of HTLV-1-specific clones was more prominent in patients with COVID-19 compared to HCs. These findings align with previous studies that reported the detection of opportunistic viral DNA reactivations in patients with COVID-19 (43–45).

## Discussion

In the present study, we comprehensively profiled the peripheral TCR β repertoire data of 192 patients with COVID-19 with different levels of disease severity and compared them with those of 81 HCs using a high-throughput sequencing approach. Our study aimed to shed light on the T-cell immune response and clonal dynamics associated with COVID-19, providing valuable insights into disease pathogenesis and potential diagnostic and therapeutic strategies.

Consistent with previous studies, we observed a significant decrease in TCR clone diversity in patients with COVID-19 compared to HCs (21, 24, 46, 47). This reduced diversity suggests a more focused and restricted T-cell response in COVID-19 patients, potentially due to the specific targeting of SARS-CoV-2 antigens. Interestingly, we found that the overall abundance of dominant clones was significantly increased in patients with COVID-19, particularly in those with more severe disease, indicating clonal expansion and enrichment of specific TCR clones. Moreover, the distribution of CDR3 sequence length showed a bias toward longer clonotypes in COVID-19-specific risk clones, which supports previous findings that antiviral TCR β CDR3s tend to be longer than autoreactive clonotypes (48).

We further investigated the presence of shared TCR clonotypes among patients with COVID-19. Contrary to the expectation that shared clonotypes would be prevalent, we found that the majority of TCR clones were private among samples, indicating significant heterogeneity in the TCR repertoires of COVID-19 patients (23, 47). This heterogeneity suggests that TCR repertoires may reflect disease severity and could potentially serve as valuable tools for immunodiagnosis. Indeed, using the identified SARS-CoV-2-associated TCRs, we achieved perfect accuracy in classifying patients with COVID-19 from HCs, demonstrating the potential of TCR analysis as a diagnostic assay based on high-throughput sequencing of peripheral blood. Additionally, our classifiers showed decent performance in distinguishing patients with different severity levels of COVID-19, except for the challenge of differentiating severe and critical cases due to their similar clinical manifestations and potential immune impairment.

We also explored the distribution of V and J gene segments and VJ pairings in patients with COVID-19. We identified several gene segments and pairings that exhibited significantly higher frequencies in patients compared to HCs, suggesting their potential involvement in the immune response against SARS-CoV-2. Notably, published SARS-CoV-2-associated TCRs had low publicity and clonal frequencies, which may be attributed to various factors, including the high heterogeneity of COVID-19-specific clones, antigenic TCR privacy, cross-reactivity with different antigens, and different HLA backgrounds (34, 49). The interplay between HLA molecules and TCRs is crucial in shaping the immune response to viral infections, and further studies exploring the correlation between HLA and TCR are warranted for future vaccine design and identifying at-risk populations.

To investigate potential connections between COVID-19 and other diseases, we analyzed the annotation of clonotypes using immune receptor databases. Interestingly, we observed a significant enrichment of T cells targeting EBV and HTLV-1 in patients with COVID-19. These findings suggest possible viral coinfections, molecular mimicry, bystander activation, and viral superantigens contributing to cross-activation and proliferation of T cells, as well as enhanced cytokine production (50–53). Notably, EBV coinfection has been associated with increased inflammation and COVID-19 severity (44). These observations highlight the complexity of immune responses during COVID-19 and the potential interplay between different viral pathogens.

Our study benefits from the large sample sizes of Chinese cohorts, providing increased statistical power and enhancing our understanding of T-cell immunity in COVID-19 (54). Furthermore, the recruitment of patients during the early stage of the disease, before the availability of vaccines or immunological agents, and sampling of HCs prior to the outbreak of COVID-19 ensure reliable comparisons and provide an original characterization of TCR repertoires under SARS-CoV-2 infection without the interference of immunotherapy and vaccines. These findings have implications for diagnostic assays, prognostication, and monitoring disease progression in COVID-19.

However, certain limitations should be acknowledged. The high mutation rates of RNA viruses, including SARS-CoV-2, necessitate concomitant adaptations across the immune system to cope with novel variants (55, 56). Therefore, our study results may not directly apply to patients with COVID-19 caused by current or potential variants. Additionally, the limited sample sizes and ethnic diversity of our population may introduce potential bias and limit the generalizability of our findings. We also acknowledge that our research methodology focused primarily on TCR sequencing and did not integrate other omics technologies, such as single-cell transcriptomics. The absence of HLA typing data further limits the validation of SARS-CoV-2-specific TCR clonotypes. Future studies should address these limitations to provide a more comprehensive understanding of adaptive immune responses against viral infections.

In summary, our study provides valuable insights into the T-cell immune response and clonal dynamics in patients with COVID-19. The decreased TCR clone diversity, specific clonal expansion, and heterogeneity of TCR repertoires observed in COVID-19 patients highlight the importance of T-cell immunity in disease pathogenesis and potential diagnostic applications. Our findings contribute to the growing body of knowledge on the adaptive immune response to SARS-CoV-2 and have implications for the development of immunotherapies, vaccines, and diagnostic tools. Future research incorporating multi-omics approaches, larger and more diverse cohorts, and HLA typing will further advance our understanding of the complex immune responses and guide clinical practice in the context of COVID-19.

## Conclusions

We uncovered distinct TCR β repertoires and SARS-CoV-2-specific TCR β clonotypes in patients with COVID-19, highlighting the heterogeneity of T cell responses and the potential of TCR β repertoires as indicators of disease severity. Our findings contribute to the growing knowledge of T cell immunity in COVID-19 and provide valuable TCR β repertoire data that can aid in understanding the immune response to SARS-CoV-2. These insights may have implications for monitoring disease progression and developing targeted interventions. Further research is warranted to decipher the intricate immune responses to SARS-CoV-2 and leverage this knowledge for effective management and prevention of COVID-19.

## Data availability statement

The datasets presented in this article are not readily available because of ethical and privacy restrictions imposed by the confidentiality agreement governing the sample information of this research. Requests to access the datasets should be directed to yangyjfw@126.com.

## Ethics statement

The studies involving human participants were reviewed and approved by The Ethical Review Board of Peking University Third Hospital approved the study protocol in March 2020, with the informed consent of participants being exempted (IRB00006761-M2020083). Written informed consent for participation was not required for this study in accordance with the national legislation and the institutional requirements.

## Author contributions

Xia-XL, CL, J-GY, H-YH, BZ, L-MC, HH, and Z-XL were responsible for the sample collection and summary of clinical information. JX, YQ, NY and Xia-XL participated in high-throughput TCRb repertoire sequencing. JX, NY, FL, G-CL, Xin-XL, YQ and C-RD contributed to bioinformatics analysis. Y-JY, Q-GG, FL, C-RD, CL, NY, Xia-XL, Y-DW, MC and JX were responsible for conceptual design, data analysis, manuscript writing, and submission. All authors contributed to the article and approved the submitted version.

## Supplementary material

Figure S1 Analysis of the clinical indicators of all patients with COVID-19. **(A)** Age and sex distribution of the four sample groups. **(B)** Risk prediction using the Cox regression model based on age index**. (C)** Risk prediction using the Cox regression model based on sex and age. **(D, E)** Kaplan–Meier analysis of the overall survival of patients with COVID-19 stratified based on IL-6 and IL-2R. **(F)** Scatter plot of the distribution of IL-2R and IL-6 in 167 patients with COVID-19.

Figure S2 Heterogeneity of the T cell receptor (TCR) β repertoires in the COVID-19 and healthy control (HC) groups. **(A)** Trend of changes in the number of clones under different detection sample numbers for each group. **(B)** Relationship between quantiles and sequencing abundance in all sample groups. **(C, D)** Proportion of overlapping clones between two samples in terms of clonotypes and abundances.

Figure S3 ROC curves showing the classification performance of the different models for the four groups using the V gene and J gene *via* 10-fold cross-validation.

Figure S4 Correlation coefficients of IL and COVID-19-associated clones in the samples.

Figure S5 Identification of clones in COVID-19 T cell receptor (TCR) β repertoire databases.
