# Supplementary figures and images for: T cell receptor β repertoires in patients with COVID-19 reveal disease severity signatures
Source: Front Immunol. 2023 Jul 5;14:1190844. doi: 10.3389/fimmu.2023.1190844 (PMC10355153; doi:10.3389/fimmu.2023.1190844)

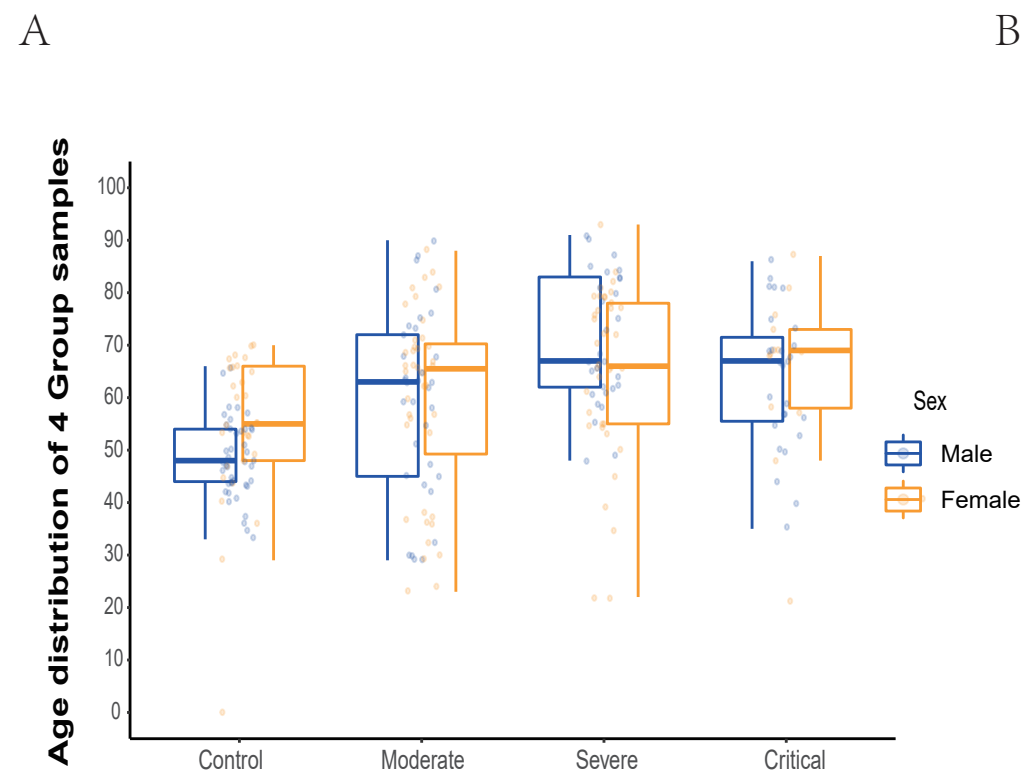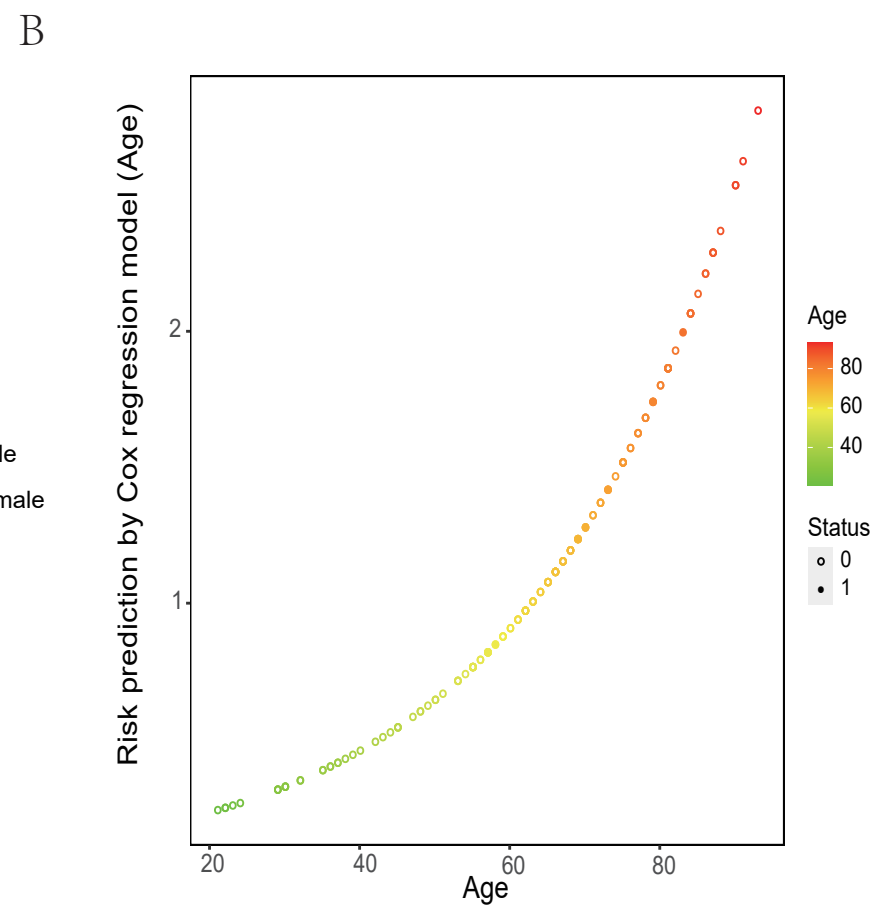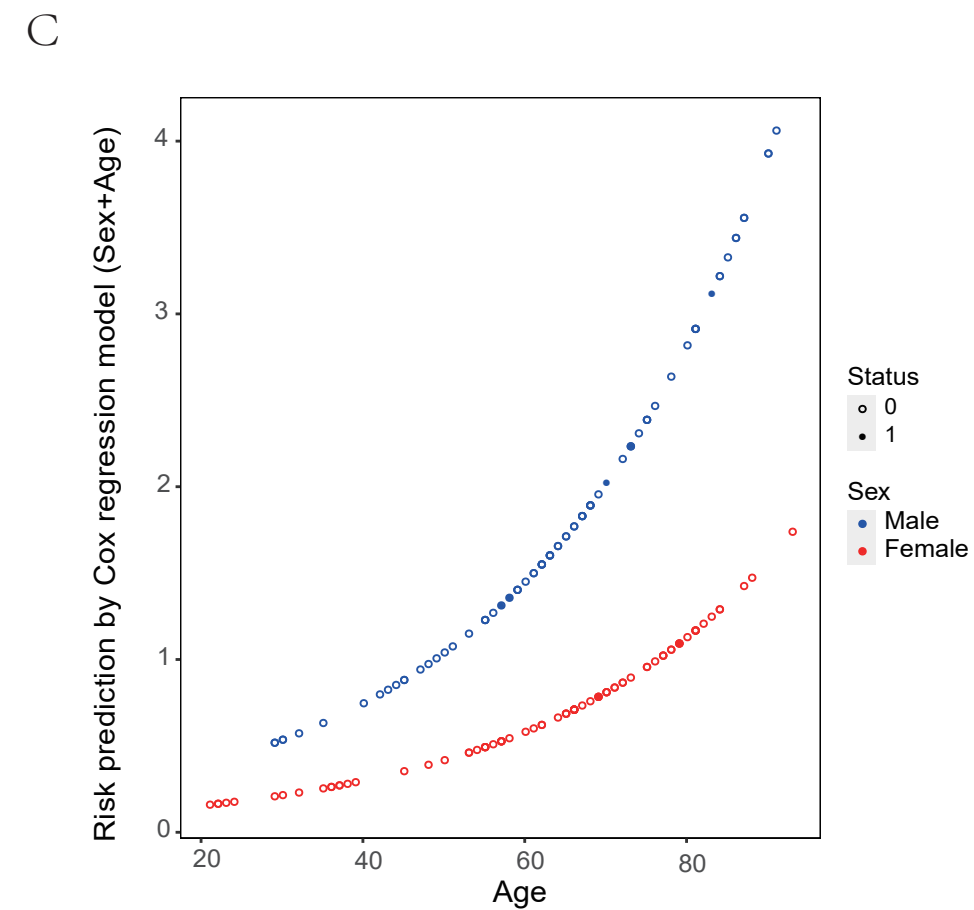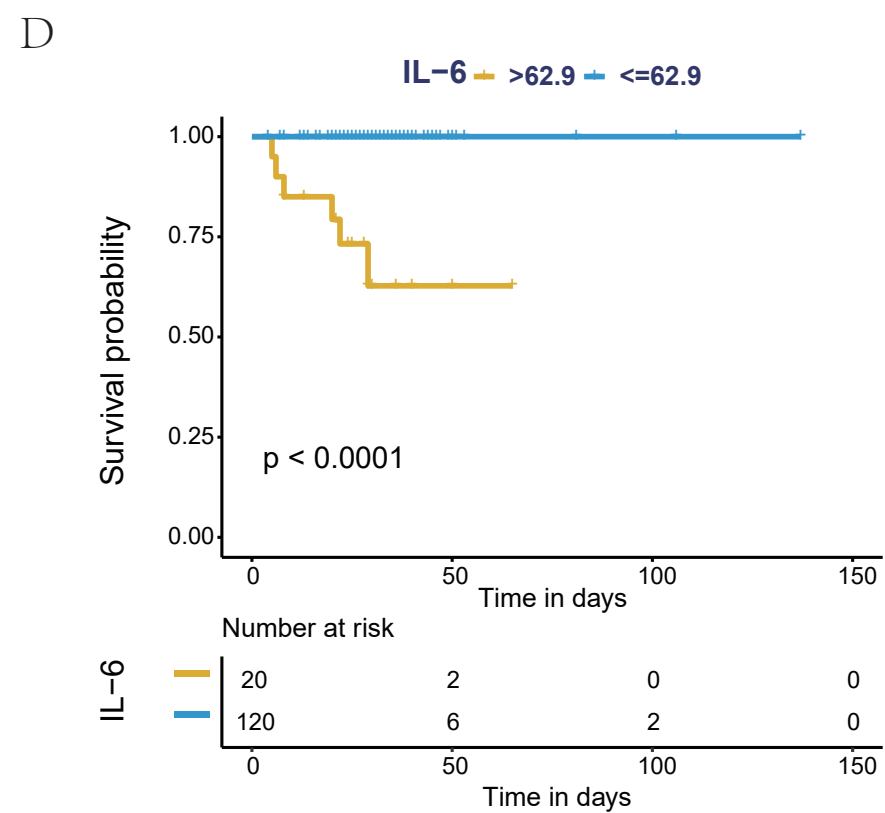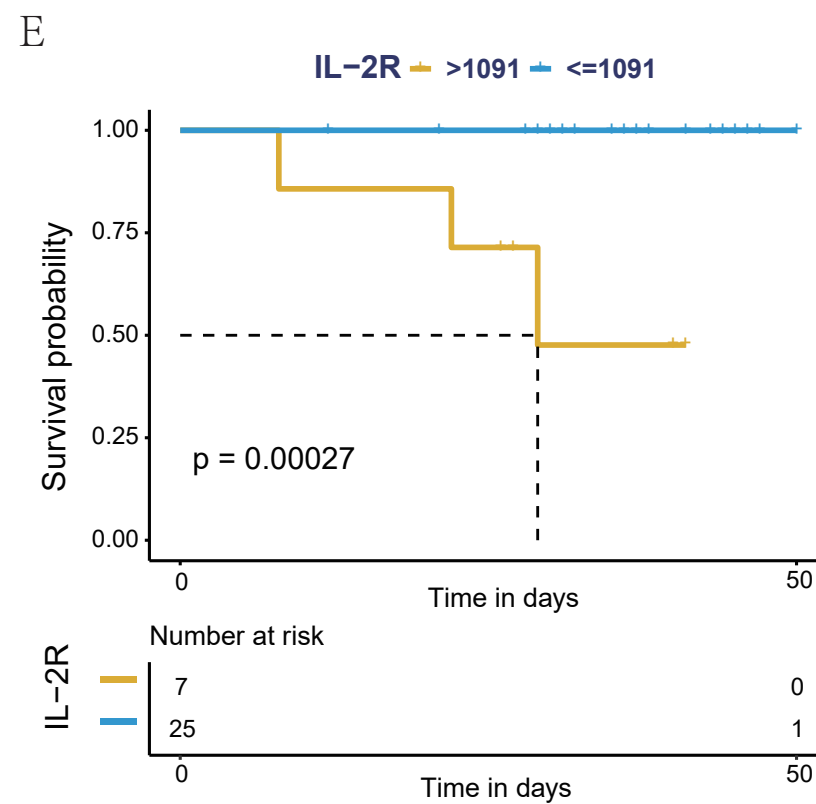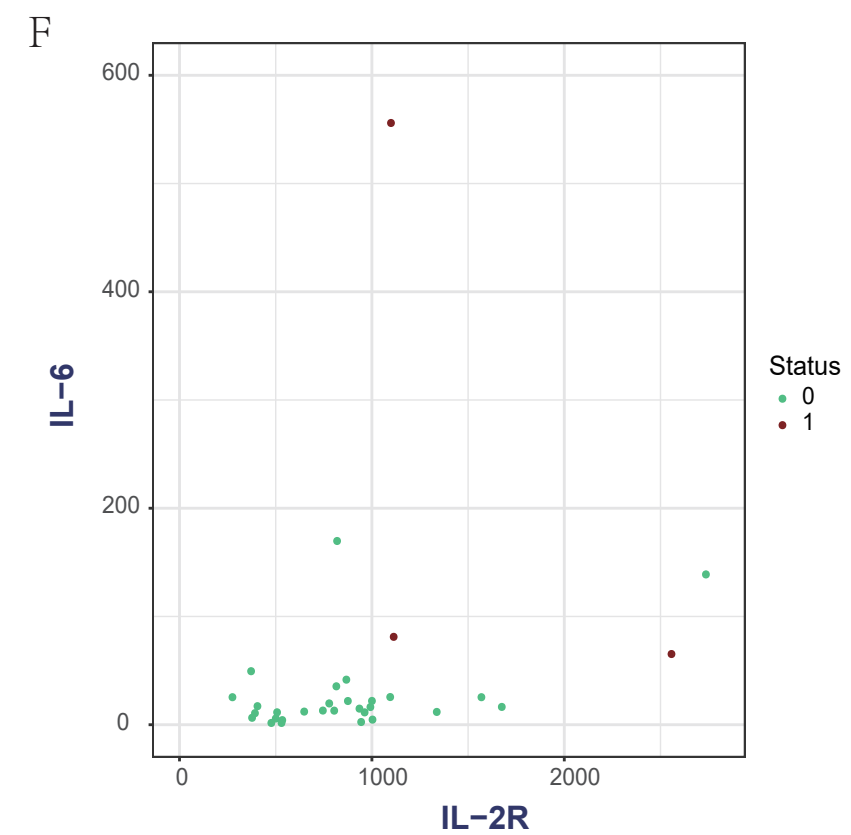

Supplement: Figure S1 — Analysis of the clinical indicators of all patients with COVID-19. (A) Age and sex distribution of the four sample groups. (B) Risk prediction using the Cox regression model based on age index. (C) Risk prediction using the Cox regression model based on sex and age. (D, E) Kaplan–Meier analysis of the overall survival of patients with COVID-19 stratified based on IL-6 and IL-2R. (F) Scatter plot of the distribution of IL-2R and IL-6 in 167 patients with COVID-19. [file Image_1.pdf]

A

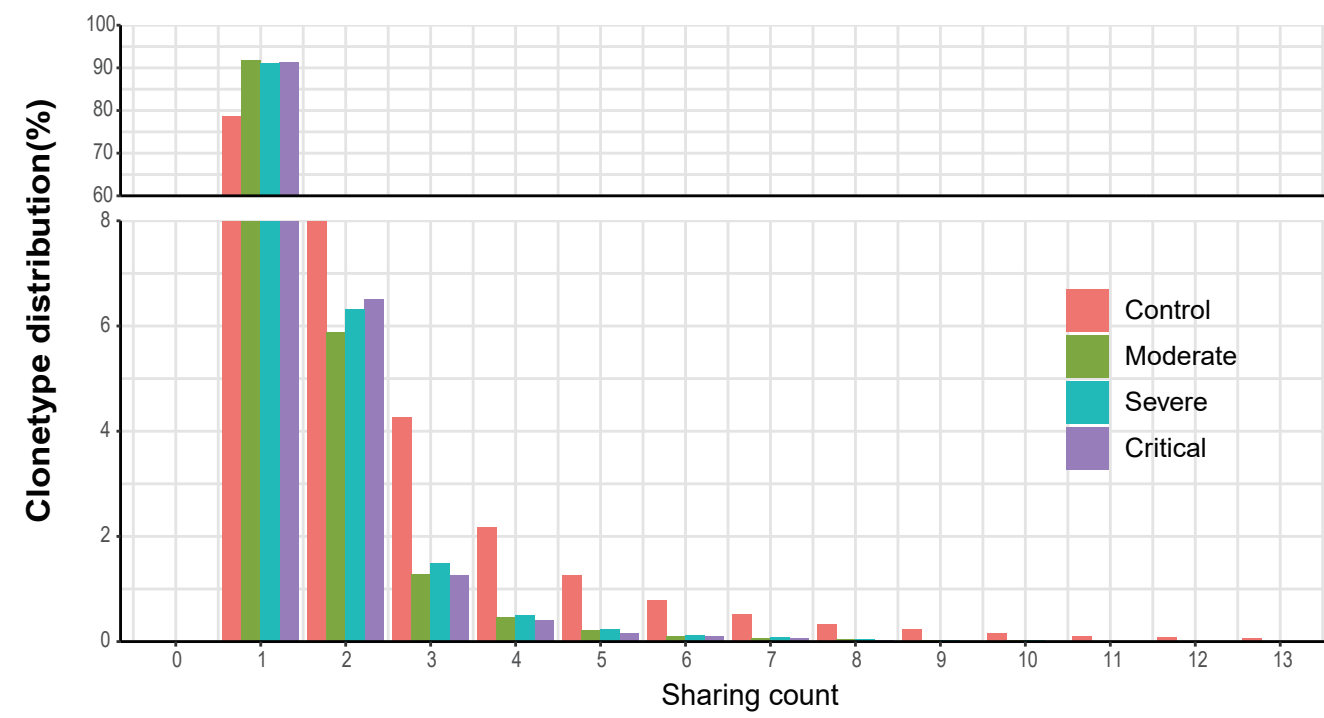

B

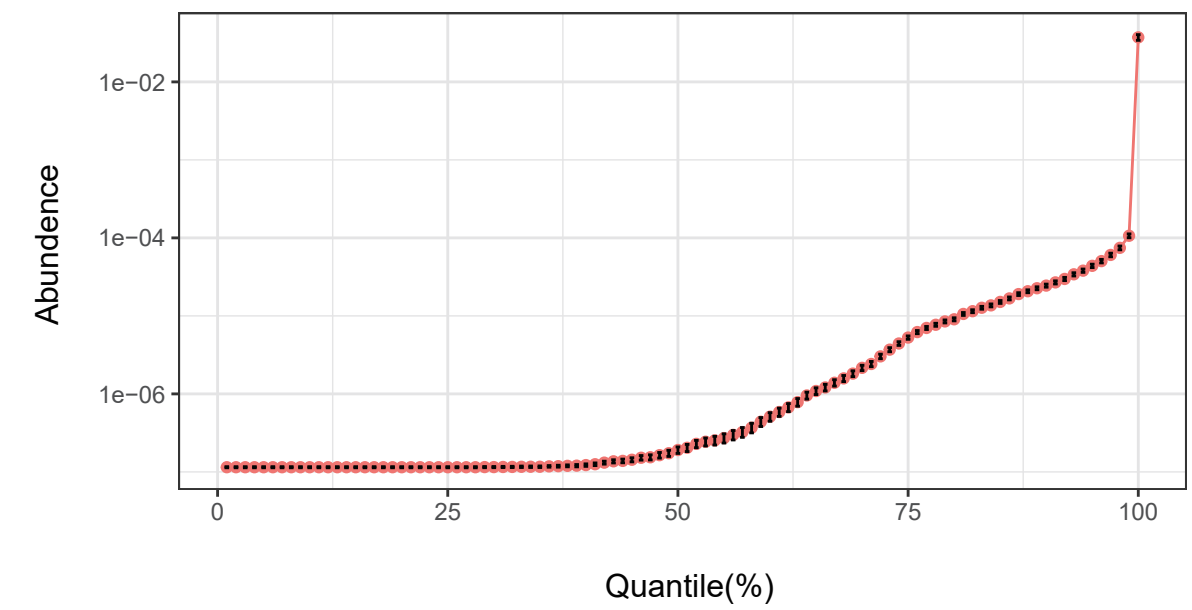

C

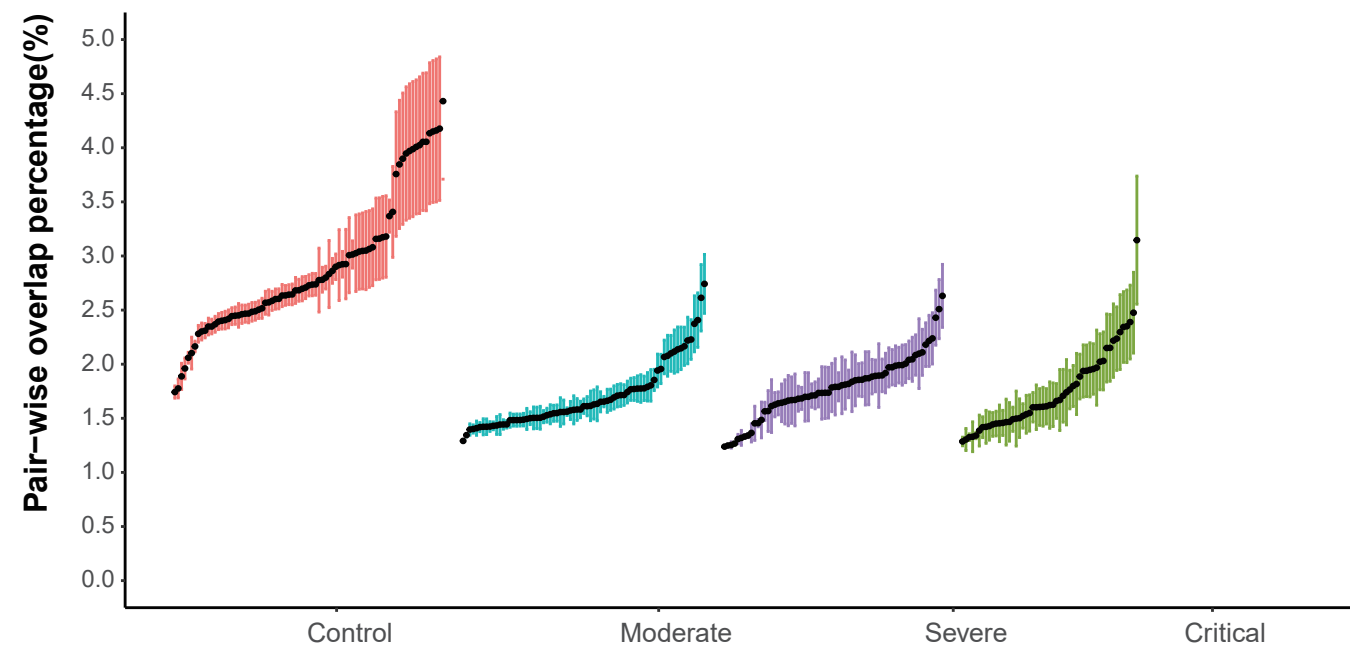

D

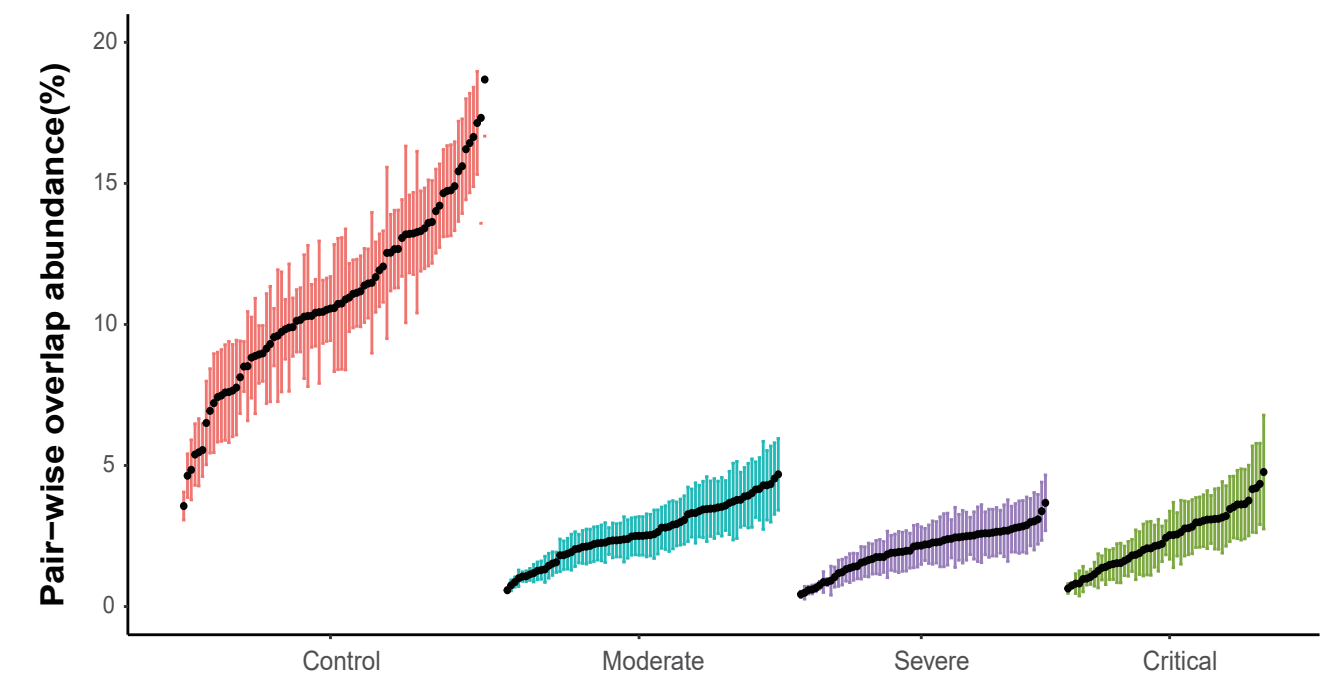

Supplement: Figure S2 — Heterogeneity of the T cell receptor (TCR) β repertoires in the COVID-19 and healthy control (HC) groups. (A) Trend of changes in the number of clones under different detection sample numbers for each group. (B) Relationship between quantiles and sequencing abundance in all sample groups. (C, D) Proportion of overlapping clones between two samples in terms of clonotypes and abundances. [file Image_2.pdf]

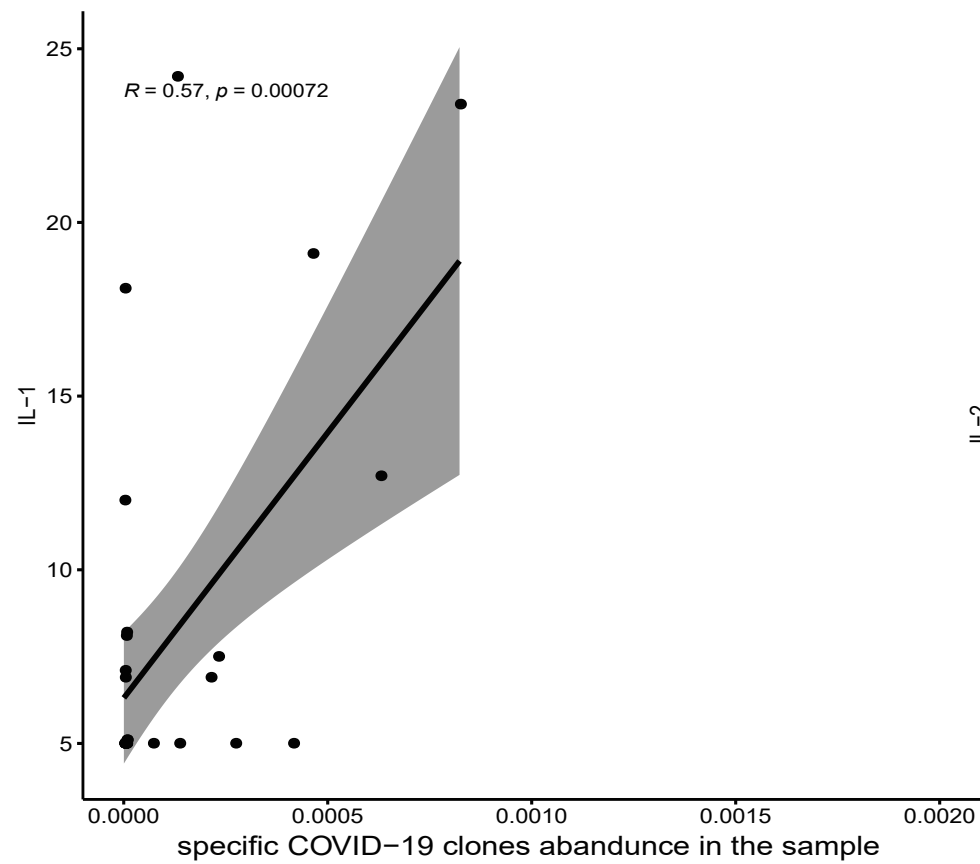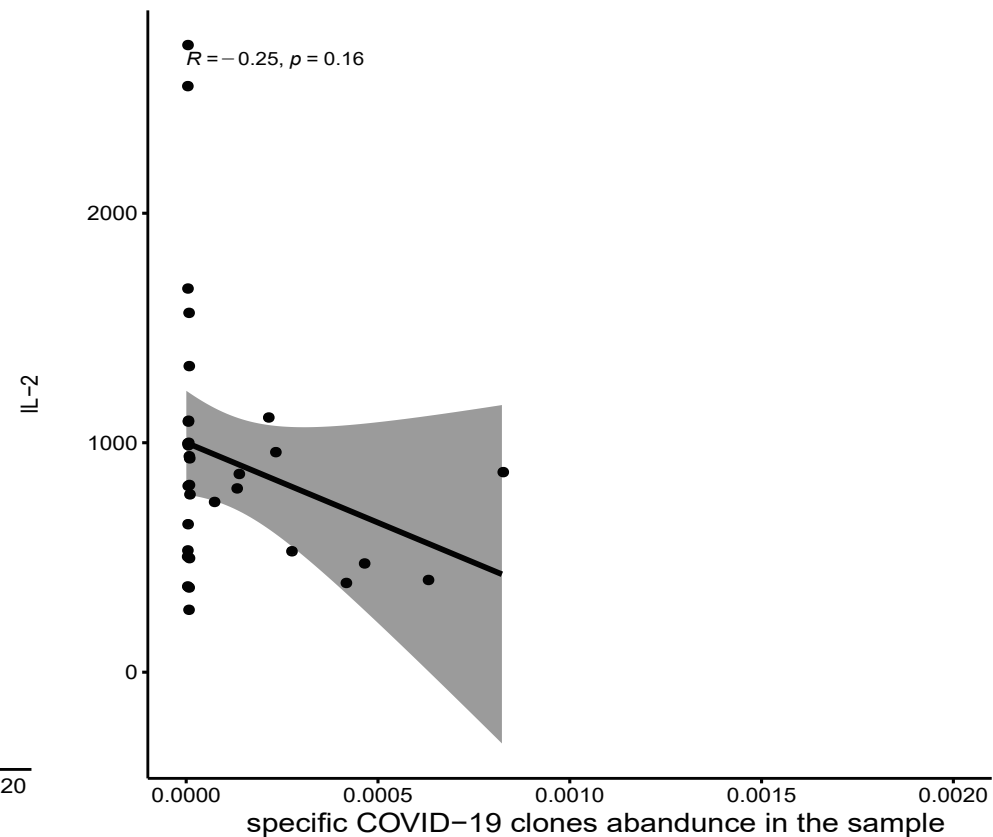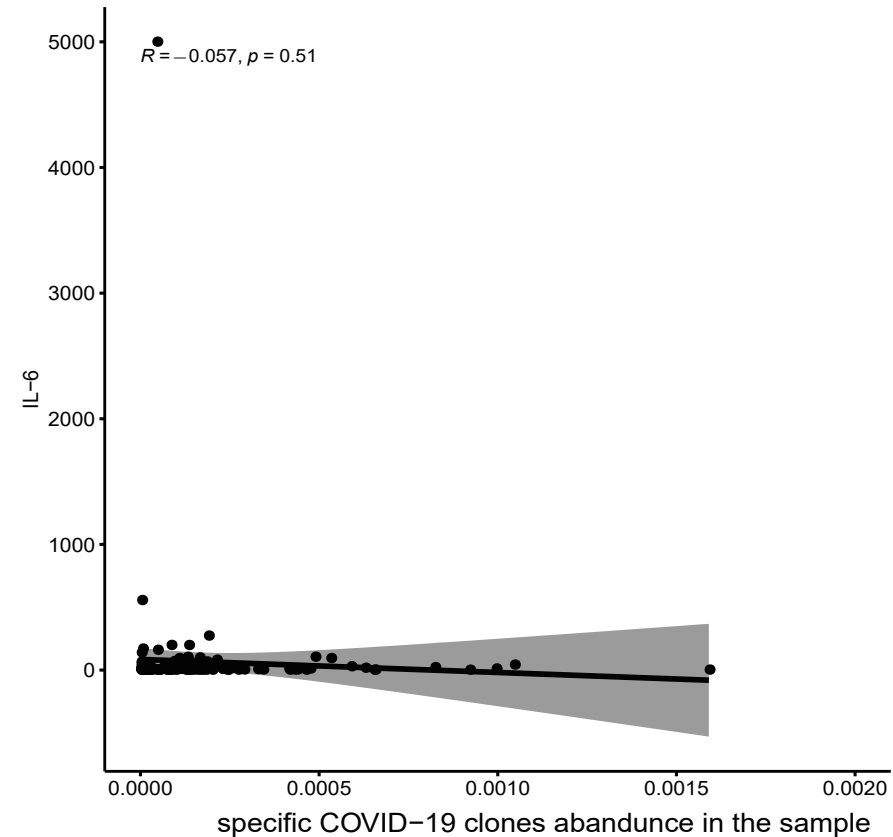

Supplement: Figure S4 — Correlation coefficients of IL and COVID-19-associated clones in the samples. [file Image_4.pdf]

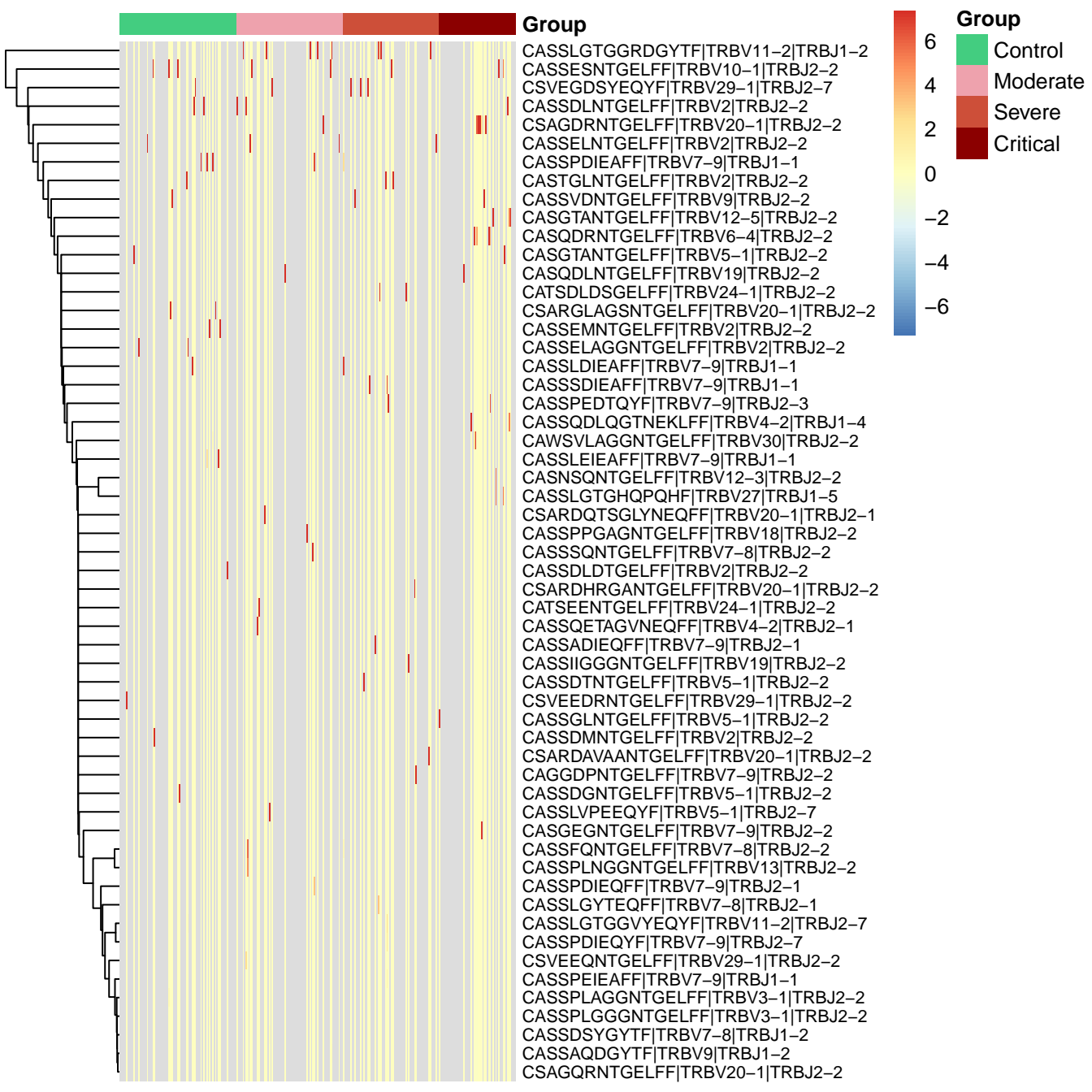

Supplement: Figure S5 — Identification of clones in COVID-19 T cell receptor (TCR) β repertoire databases. [file Image_5.pdf]
