# Supplementary material for: T cell receptor β repertoires in patients with COVID-19 reveal disease severity signatures
Source: Front Immunol. 2023 Jul 5;14:1190844. doi: 10.3389/fimmu.2023.1190844 (PMC10355153; doi:10.3389/fimmu.2023.1190844)
Supplement: Figure S3 — ROC curves showing the classification performance of the different models for the four groups using the V gene and J gene via 10-fold cross-validation. [file Image_3.pdf]

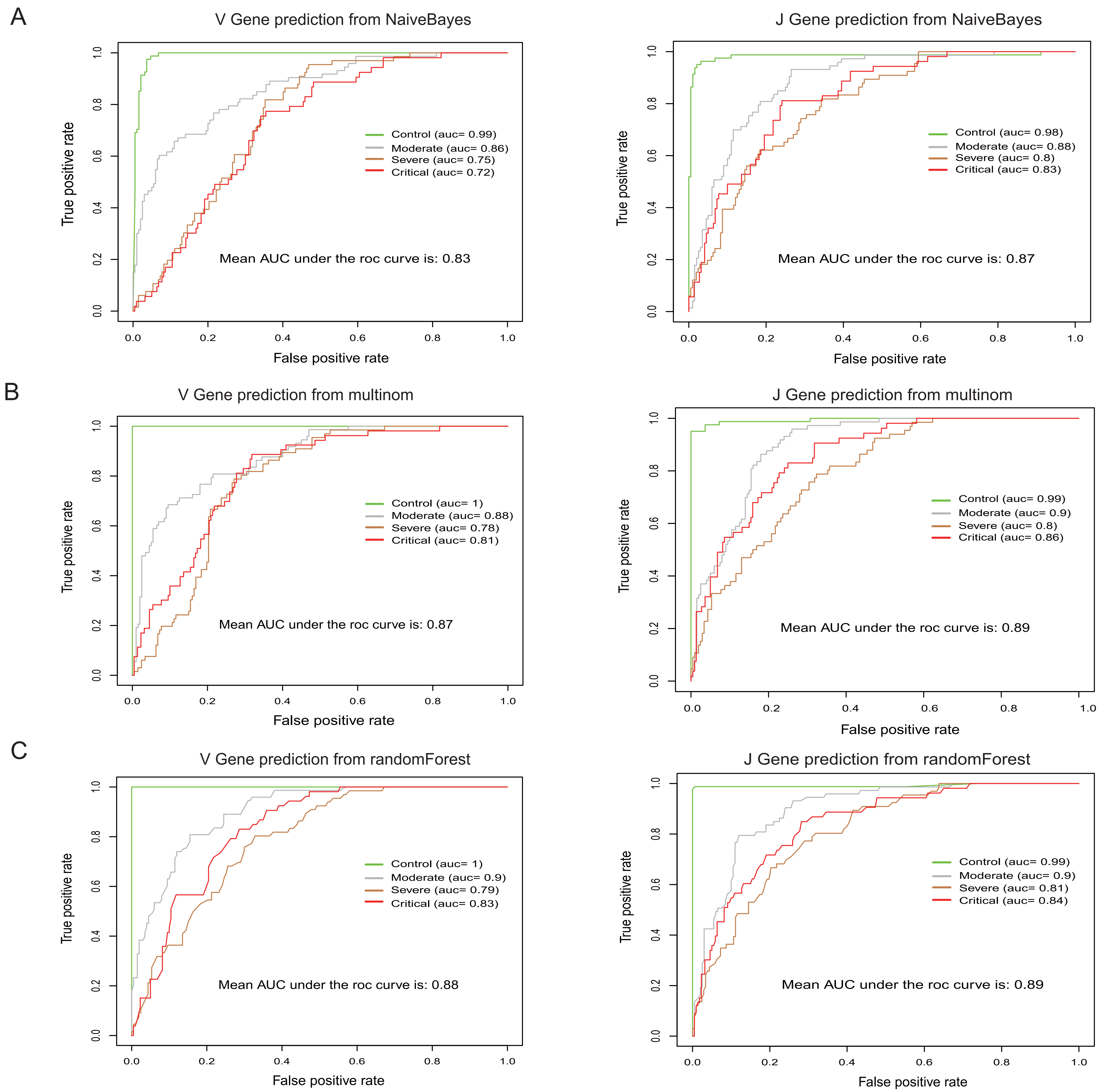
A
V Gene prediction from NaiveBayes
Control (auc= 0.99)
Moderate (auc= 0.86)
Severe (auc= 0.75)
Critical (auc= 0.72)
Mean AUC under the roc curve is: 0.83
True positive rate
False positive rate
J Gene prediction from NaiveBayes
Control (auc= 0.98)
Moderate (auc= 0.88)
Severe (auc= 0.8)
Critical (auc= 0.83)
Mean AUC under the roc curve is: 0.87
True positive rate
False positive rate
B
V Gene prediction from multinom
Control (auc= 1)
Moderate (auc= 0.88)
Severe (auc= 0.78)
Critical (auc= 0.81)
Mean AUC under the roc curve is: 0.87
True positive rate
False positive rate
J Gene prediction from multinom
Control (auc= 0.99)
Moderate (auc= 0.9)
Severe (auc= 0.8)
Critical (auc= 0.86)
Mean AUC under the roc curve is: 0.89
True positive rate
False positive rate
C
V Gene prediction from randomForest
Control (auc= 1)
Moderate (auc= 0.9)
Severe (auc= 0.79)
Critical (auc= 0.83)
Mean AUC under the roc curve is: 0.88
True positive rate
False positive rate
J Gene prediction from randomForest
Control (auc= 0.99)
Moderate (auc= 0.9)
Severe (auc= 0.81)
Critical (auc= 0.84)
Mean AUC under the roc curve is: 0.89
True positive rate
False positive rate
